# Making a reality of social and health care integration: lessons from the Italian peninsula

**DOI:** 10.5334/ijic.806

**Published:** 2011-09-30

**Authors:** Paolo Pasini

**Affiliations:** General Director of the Institute for Health and Social Welfare, Republic of San Marino

It is often maintained that integrated care is the only approach that can produce good health care whilst simultaneously improving a system's accountability to social care needs. In this sense, integrated care defines a context in which ‘caring’ means ‘taking care’—in other words, the process whereby members of the public are provided with complete assistance involving an integrated path amid primary, specialist, hospital and social assistance. In particular, the degree of integration between health and social care is a crucial factor that influences the effectiveness of a person's ability to navigate the complex web of care they need and overcome the fragmentation and inefficiency of social-health systems.

The problem of social and health care integration has been very often addressed in contributions and articles to IJIC [1, 2]. In this Editorial, we reflect on this evidence and the recurring problems regarding the development of integrated care at a local level. What comes through very clearly is that the problems of integration are, more or less, constant and recurrent when compared across different care systems—for example, in the common dichotomy between health services that are provided by the central State or Regions and social services, nearly always provided by the Municipalities or private health concerns.

The problem is not only that ‘Social’ and ‘Health’ often differ in themselves in terms of purposes and methods, but that the very public and private bodies which provide the services have very different missions, operating systems, and management and institutional logics. In view of this, it is hard to conceive an effective integrated mode of operation. On the contrary, care systems most often seek to differentiate, rather than assist in the integration of, these activities, for example to avoid duplication of tasks involving the various providers. At administrative level, too, there are often big difficulties: lack of shared budgets, decision-making bodies that are not linked, service systems that are totally different and operate with separate rules of governance and accountability. Hence, we often assist in a sort of ‘institutional rejection’ on the part of users and the general public who regard the system as ‘unhelpful’ in response to their needs.

Of course, we have painted a rather ‘dim’ picture of the situation and made circumstances look worse in order to better exemplify operating conditions. But problems of integration are persistent and risk thwarting the efforts made inside the health system to provide integrated care. We think, therefore, that it would be useful to spend a word or two on how the Italian peninsula has sought to address this problem, drawing especially from the progress made in the Emilia Romagna Region and the Republic of San Marino.

In Emilia Romagna, there is a clear distinction between Health Assistance and Social Assistance. However, during the course of recent service revamping, the Region has granted more powers to local bodies by setting up the ‘*Social and Health District*’ within Local Health Units (public organisations formed on a territorial basis that coincides with a Provincial district). The *Social and Health District* is the place where Municipal and Local Health Unit planning is integrated, where operating programmes are drawn up, and where common goals for services are discussed and planned for key client groups such as the elderly, cancer patients, minors, drug users and people with mental health problems.

As a result of this work the Municipalities, the local Health Units and the Third Sector (private and voluntary) make agreements to supply integrated services, pooling personnel and resources. The Social and Health Districts then assess results compared to the changes formulated as necessary for the intervention programmes developed.

As regards San Marino, social and health care is more organically integrated. San Marino is the oldest surviving Republic in the world (founded in the age of Diocletian in the year 304 A.D.) and was founded as a monastic community. This historical context is important since the need for care continuity and integration of health and well-being between social assistance and health care is part of the cultural (and therefore political-technical) DNA of this small community of 30,000 people. In San Marino, social, health and social-security services are provided by a single body—the *Social-Security Institute*. Consequently, institutional integration enables easier planning and provision of social and health services when compared to other countries where divisions between municipalities, regions and the state create major organisational and regulatory problems.

In San Marino, social-health service integration is achieved at both operation and professional level. Crucially, such an approach is advocated and embedded in its first ‘social-health plan’ of 2006 in which the main points concerning the characteristics of integration between social and health services were set out. This plan has sought to overcome parochial attitudes and to integrate different skills and services through the singleness and totality of intervention. This is based on the view that it is only by involving the different interlinked activities through complex assistance processes that it is possible to satisfy care needs that require, at the same time, both health services and social protection measures. The aim is to ensure the continuity of care and rehabilitation services, including in the long-term. In order to trigger and embed this type of change operationally, the following aspects are taken into consideration:

- individual and group accountability, in the case of integrated services, for the quantity and the appropriateness of the available services;
- overcoming parochial outlooks, focused on individual professional interests;
- flexible use of human resources, without tying them down to pre-defined places, times and fields of action.

It is interesting to note how the Social-Security Institute has just one budget for social and health services. The organisation does not contemplate the hierarchical separation of the two sectors (social and health) and per capita expenditure is low (for 2010 this did not exceed €1720, despite life expectancy in San Marino being among the highest in the world). The link between the cost-effectiveness of the care system in San Marino and its relationship to its integration of health and social services at a local level needs to be investigated. Nevertheless, we think San Marino's experiences cannot but prompt us to suggest the more widespread experimentation of integrated health and social care in larger territorial contexts.

For all of you who are interested in making a reality of health and social care integration, and in learning more about the case of San Marino in particular, we invite you to attend the 12th International Conference on Integrated Care to be held in the Republic of San Marino on March 29–30, 2012 (http://www.integratedcare.org/).
